# Extrauterine Low-Grade Endometrial Stromal Sarcoma: A Case Report and Review of the Literature

**DOI:** 10.3390/diagnostics16142157

**Published:** 2026-07-10

**Authors:** Jie-Yu Li, Chiu-Hsuan Cheng, Dah-Ching Ding

**Affiliations:** 1 Department of Obstetrics and Gynecology, Hualien Tzu Chi Hospital, Buddhist Tzu Chi Medical Foundation, Tzu Chi University, Hualien 970, Taiwan; twilighter530@gmail.com; 2 Division of Digital Pathology, Department of Anatomical Pathology, Hualien Tzu Chi Hospital, Buddhist Tzu Chi Medical Foundation, Hualien 970, Taiwan; chiuhsuan.cheng@gmail.com; 3 Department of Pathology, School of Medicine, Tzu Chi University, Hualien 970, Taiwan; 4 Institute of Medical Sciences, Tzu Chi University, Hualien 970, Taiwan

**Keywords:** low-grade endometrial stromal sarcoma, extrauterine endometrial stromal sarcoma, immunohistochemistry, progesterone therapy, rare gynecologic malignancy

## Abstract

**Background and Clinical Significance**: The primary occurrence of low-grade endometrial stromal sarcoma (LG-ESS) at extrauterine sites, termed extrauterine ESS (EESS), is exceedingly rare. **Case Presentation**: A 49-year-old female, gravida 2 para 2 (all deliveries by cesarean section), presented with a 1-month history of epigastric and left upper quadrant abdominal pain. Abdominal computed tomography revealed a lobulated mass, measuring 4.6 cm, in the left upper quadrant with focal attachment to the splenic flexure of the colon. She underwent a laparoscopic partial gastrectomy and colectomy at a referral institution. Surgical pathology identified a 6.0 × 5.0 × 4.0 cm lobulated tumor involving the stomach with colonic invasion, diagnosed as LG-ESS (pT3N0). Immunohistochemistry showed a CD10-reactive lesion. The patient was referred to our institution for definitive gynecological surgery. After prophylactic ureteral double-J catheter insertion, she underwent laparoendoscopic single-site (LESS) total hysterectomy with bilateral salpingo-oophorectomy. The final histopathological examination of the hysterectomy specimen revealed intramural leiomyomas and benign ovarian cysts, with no evidence of ESS within the uterine corpus or adnexa. These findings confirmed a true primary EESS of nonuterine origin, consistent with AJCC stage 3 disease. The patient recovered uneventfully and was discharged during outpatient follow-up. **Conclusions**: This case represents one of the very few reported instances of primary LG-EESS involving the stomach and colon, simultaneously managed with a staged multidisciplinary surgical approach, including gastrointestinal resection followed by LESS hysterectomy and bilateral salpingo-oophorectomy.

## 1. Introduction

Endometrial stromal sarcoma (ESS), the second most common malignant uterine mesenchymal neoplasm, accounts for approximately 0.2% of all reported reproductive tract malignancies [1]. ESS is classified as low-grade ESS (LG-ESS), high-grade ESS, or undifferentiated uterine sarcoma based on its histomorphology, immunohistochemistry, and molecular characteristics [2].

LG-ESS is characterized by indolent clinical behavior, strong hormone receptor positivity, and a propensity for late recurrence, often decades post initial diagnosis [3]. Although typically arising within the uterine corpus, ESS originates from extrauterine sites, a phenomenon termed extrauterine ESS (EESS), most commonly in the setting of preexisting endometriosis [4]. EESS pathogenesis involves the malignant transformation of ectopic endometrial stromal cells, paralleling the well-established endometriosis-to-malignancy sequence observed in endometrioid and clear-cell carcinomas of the ovary [5].

Primary EESS is exceptionally rare, with only sporadic case reports and small case series published in the literature. Documented sites of origin include the ovary, omentum, peritoneum, vagina, ureter, colon, and rectum, among others [4,6,7]. A 2022 systematic review by Deb and Heller identified tumor site and mode of treatment as significant determinants of overall and progression-free survivals in patients with EESS [8], while Wu et al. (2022) reported 5- and 10-year overall survival rates of 100% in a cohort of 10 LG-EESS patients managed with cytoreductive surgery and adjuvant hormonal therapy [9]. Gastrointestinal involvement, particularly colonic and gastric involvement, is one of the rarest manifestations of EESS, and its preoperative distinction from primary gastrointestinal stromal tumors (GIST), leiomyosarcomas, or metastatic carcinomas poses a significant diagnostic challenge. Immunohistochemical profiling, including CD10, estrogen receptor (ER), progesterone receptor (PR), and beta-catenin, remains indispensable in confirming the endometrial stromal lineage of such extrauterine tumors [10].

Herein, we report the case of a 49-year-old female, gravida 2 para 2, who had all prior deliveries via cesarean section and was diagnosed with LG-ESS involving the stomach and colon. She underwent resection of the involved gastrointestinal segments at an external institution, following which she was referred to our center for definitive gynecologic surgery. Given her clinical presentation and extrauterine distribution of the disease, a laparoendoscopic single-site (LESS) approach was planned to complete a total hysterectomy and bilateral salpingo-oophorectomy to address the presumed primary uterine source and eliminate residual hormonally responsive tissue [10]. This case highlights the diagnostic complexity and multidisciplinary surgical demands of gastrointestinal LG-EESS and emphasizes the importance of minimally invasive gynecologic surgery for the comprehensive management of this rare malignancy [11].

## 2. Case

### 2.1. Ethics

The patient provided written informed consent for the publication of this case report, including all clinical data and images.

### 2.2. Chief Complaint

Epigastric pain and LUQ abdominal pain for 1 month.

### 2.3. Case Report

Our patient, a 49-year-old female, gravida 2, para 2 (all deliveries by cesarean section), presented with a 1-month history of epigastric pain and left upper quadrant (LUQ) abdominal pain. She also complained of intermittent lower back throbbing pain, progressive abdominal fullness, and increased urinary frequency. No dysuria, unintentional weight loss, nausea, vomiting, or fever was noted. Her menstrual history was notable for dysmenorrhea and menorrhagia, with her last menstrual period occurring on 29 March 2026. The patient’s active menstrual cycle confirmed premenopausal status at the time of presentation, which is clinically relevant in the context of LG-ESS given the well-established estrogen-driven biology of this tumor. In premenopausal women, endogenous ovarian estrogen production provides a continuous mitogenic stimulus to ER- and PR-positive tumor cells.

The relevant medical history included a right internal carotid artery (ICA) ophthalmic segment aneurysm (dome/neck ratio 2.0/2.2 mm), autoimmune thyroiditis in a euthyroid state (anti-TPO 221.6, anti-TG 21.6), and gastroesophageal reflux disease (GERD). Her past medical history included three comorbidities of perioperative relevance: a right ICA ophthalmic segment aneurysm, deemed low-risk for rupture under controlled general anesthesia with careful blood pressure management. Autoimmune thyroiditis in a confirmed euthyroid state, requiring no specific perioperative intervention. GERD, which informed the decision to perform rapid sequence induction to minimize aspiration risk during intubation.

Her surgical history included transcervical resection myomectomy for submucosal leiomyoma in March 2021 and left medial meniscus repair in December 2022. She reported allergies to tetracycline (lip swelling and numbness), doxycycline, diclofenac, and some muscle relaxants.

On 19 March 2026, with progressive worsening of LUQ pain, she was evaluated at the emergency department of another hospital. Standing kidneys-ureters-bladder (KUB) and bedside ultrasonography findings were unremarkable. Abdominal computed tomography (CT) revealed a lobulated mass lesion between the LUQ of the abdomen (gastric greater curvature) and the splenic flexure of the colon, measuring at least 4.6 cm (Figure 1), and prominent left ovarian vein and parauterine vessels.

At the time of initial presentation, the primary differential diagnoses considered for the left upper quadrant abdominal mass with splenic flexure colonic attachment included gastrointestinal stromal tumor (GIST), primary colorectal adenocarcinoma, leiomyosarcoma, and metastatic carcinoma. A gynecologic origin—including ovarian malignancy or extrauterine uterine mesenchymal tumor—was additionally entertained given the imaging finding of prominent left ovarian vein and parauterine vascularity on abdominal CT. The absence of specific imaging features pathognomonic of any single entity, combined with the unusual anatomical location bridging the stomach and colon, necessitated surgical resection with pathological confirmation for definitive diagnosis.

On 26 March 2026, the patient underwent laparoscopic partial gastrectomy and colectomy. Surgical pathology at the referring institution demonstrated a lobulated tumor involving the stomach with invasion into the colon, measuring 6.0 × 5.0 × 4.0 cm, with the tumor margin 1.8 cm from the gastric resection margin. The pathological diagnosis was low-grade endometrial stromal sarcoma (LG-ESS), pT3N0 (Figure 2). Immunohistochemical analysis of paratubal tissue revealed CD10-positive and Desmin-negative lesions (Figure 3). Estrogen and progesterone receptors (ER and PR) were positive for staining. The patient was subsequently referred to our institution for further gynecological management.

Following referral to our institution, preoperative abdominal and pelvic imaging was reviewed and confirmed postoperative changes from the prior partial gastrectomy and partial colectomy, with no evidence of residual intra-abdominal disease or pathological lymphadenopathy. However, a dedicated chest CT for pulmonary staging was not performed prior to hysterectomy, which, in retrospect, represents an incomplete oncologic staging workup for a confirmed malignant mesenchymal neoplasm.

Upon admission to our department, vital signs were stable (blood pressure 112/70 mmHg, pulse 77 bpm, temperature 36.3 °C, respiratory rate 16/min). Preoperative laboratory workup revealed a hemoglobin level of 11.8 g/dL, white blood cell counts of 3.92 × 10^3^/μL, platelet count of 262 × 10^3^/μL, and an INR of 0.97. Renal and hepatic function test results were within normal limits. Before the planned surgery, urologists were consulted for prophylactic double-J ureteral catheter insertion. On 15 April 2026, she underwent LESS total hysterectomy with bilateral salpingo-oophorectomy (BSO). Intraoperative findings revealed an enlarged uterus with a myoma and normal-appearing bilateral adnexa (Figure 4). Adhesions were observed at the umbilical wound region from the prior gastrointestinal surgery. The total operative time was 101 min, with an estimated blood loss of 300 mL. No blood transfusion was required, and no intraoperative complications occurred.

Final surgical pathology of the hysterectomy specimen (uterus measuring 11.5 × 8.0 × 5.0 cm) demonstrated intramural leiomyomas (up to 1.5 cm), chronic cervicitis with Nabothian cysts, a right follicular ovarian cyst, and a left corpus luteum cyst with epithelial inclusion cysts. Critically, no LG-ESS were identified within the uterine corpus or adnexa.

Immunohistochemical analysis of the paratubal tissue at our institution showed no CD10-reactive lesion; CD117 highlighted scattered mast cells, desmin-marked smooth muscle fibers, and tubal epithelial cells were ER- and PR-positive. These findings confirmed that the previously resected gastrointestinal tumor represented a primary extrauterine LG-ESS without uterine involvement, consistent with primary EESS. As no validated disease-specific staging system exists for primary EESS, the AJCC/TNM staging system for soft tissue sarcomas of the abdomen and thoracic visceral organs was applied analogously. The resected gastrointestinal tumor, measuring 6.0 × 5.0 × 4.0 cm with direct invasion into the adjacent colon, was classified as pT3N0, corresponding to AJCC Stage III soft tissue sarcoma. FIGO staging for uterine sarcomas was considered inapplicable given the confirmed absence of uterine involvement on final hysterectomy pathology.

The patient recovered uneventfully and was discharged in a stable condition for outpatient follow-up of 2 months. Following surgical resection, the patient was treated with high-dose progesterone as adjuvant hormonal therapy.

## 3. Discussion

### 3.1. Overview of Reported Cases

The present case adds to the growing yet sparse body of literature on primary LG-EESS. As summarized in Table 1, we reviewed 27 references comprising case reports, case series, and systematic and observational studies published between 1992 and 2025. The reported cases spanned a wide age range (20–80 years), with most patients in the perimenopausal or premenopausal period. Sites of extrauterine involvement are remarkably heterogeneous and include the omentum, ovary, peritoneum, vagina, colon, sigmoid colon, rectovaginal septum, vulva, ureter, jejuno-ileum, and, as in the present case, the stomach and transverse colon, one of the rarest gastrointestinal presentations documented to date [3,8,12,13,14]. Endometriosis is a concurrent or predisposing condition in a substantial proportion of reported cases [1,5,9,11,15,16,17,18,19,20,21,22], consistent with the prevailing hypothesis that the ectopic endometrial stroma undergoes malignant transformation. Treatment in most reported cases comprises surgical resection with or without adjuvant hormonal therapy, and the outcomes are generally favorable, particularly when optimal cytoreduction is achieved [5,8,9,10]. The systematic review by Deb and Heller [8] and observational cohort study by Dai et al. [10] represent the most comprehensive analyses to date and establish that tumor site, surgical adequacy, and ovarian status are the primary determinants of recurrence and survival. The 10-case series reported by Wu et al. demonstrated 100% 5- and 10-year overall survival, underscoring the indolent yet recurrence-prone nature of LG-EESS [9]. Taken together, the literature confirms that LG-EESS, although rare, has a prognosis comparable to that of its uterine counterpart when managed with complete surgical resection and appropriate hormonal suppression.

### 3.2. Etiology, Diagnosis, Treatment, and Prognosis

#### 3.2.1. Etiology

LG-EESS pathogenesis remains to be fully understood; however, the malignant transformation of extrauterine endometriosis is the most widely accepted mechanism [9,11,15,19,20]. Endometriosis affects approximately 10% of females of reproductive age [28] and, in a subset of cases, undergoes malignant transformation, most commonly to endometrioid or clear cell carcinoma of the ovary but rarely to ESS [18,22]. The endometriosis-to-ESS sequence is supported by histological evidence of the transition from benign endometriotic stroma to low-grade sarcoma, as demonstrated by Vierhout et al. [15], who used DNA flow cytometry to show changes in nuclear DNA content at the malignant interface. Molecular studies have identified JAZF1-SUZ12 gene fusion (resulting from t(7;17)(p15;q21) translocation) as a hallmark of LG-ESS, present in both uterine and extrauterine tumors, regardless of the endometriosis status [12,27], indicating that endometriosis may provide a permissive stromal environment for neoplastic transformation rather than being a strict prerequisite. The role of estrogen stimulation in driving tumor growth is underscored by the near-universal expression of the estrogen receptor (ER) and progesterone receptor (PR) in LG-EESS, as well as favorable responses to hormonal ablation [5,9,17,23]. In our case, the patient had a documented history of dysmenorrhea and menorrhagia, and the tumor arose at an extrauterine gastrointestinal site in the absence of an identifiable primary uterine ESS, a pattern consistent with *de novo* EESS arising from an ectopic endometrial stroma potentially seeded along the gastrointestinal serosa.

#### 3.2.2. Diagnosis

Preoperative diagnosis of LG-EESS is evolving, particularly at gastrointestinal sites where the differential diagnosis includes gastrointestinal stromal tumor (GIST), leiomyosarcoma, carcinoid tumor, and metastatic carcinoma [3,12,13,14]. The presenting symptoms are nonspecific, as illustrated in our patient, who presented with epigastric and left upper quadrant pain, and imaging studies, while useful in localizing the mass, are rarely diagnostic of stromal lineage. Abdominal CT revealed a lobulated LUQ mass with colonic attachment and prominent parauterine vascularity, features that raised suspicion of a gynecologic origin but required pathological confirmation. MRI may be more informative, as LG-EESS typically appears as a solid or solid cystic mass with an intermediate T2 signal, restricted diffusion, and heterogeneous enhancement, which, when combined with a history of endometriosis, should prompt consideration of EESS [1,18]. Haghighatkhah et al. [1] emphasized the utility of preoperative MRI for LG-EESS arising from peritoneal endometriosis. The definitive diagnosis was based on histopathology and immunohistochemistry. The characteristic morphology comprises uniform, small, oval-to-spindle cells resembling proliferative-phase endometrial stroma, with low mitotic activity and a rich vascular network of small arterioles [5].

Complete preoperative staging in LG-EESS should include chest CT to exclude pulmonary metastases, which, while uncommon, have been documented in advanced or recurrent LG-ESS and carry significant prognostic implications [8]. In the present case, chest CT was not performed prior to the definitive hysterectomy, highlighting a gap in the staging workup that clinicians should be mindful of when managing patients with confirmed EESS referred for completion surgery. We therefore recommend that chest CT be considered a standard component of the preoperative evaluation in all patients with histologically confirmed LG-EESS, in addition to abdominal and pelvic imaging, prior to any planned surgical intervention.

Immunohistochemically, LG-EESS is typically positive for CD10, ER, PR, and vimentin and negative for CD117 (c-kit), CD34, DOG1, desmin, and h-caldesmon, a profile that distinguishes it from GIST and smooth muscle tumors [1,5,16,18]. Notably, CD10 may be focally negative or lost, particularly with underfixation, as highlighted by Tang et al. [2]; in such cases, beta-catenin, ER, and PR positivity can be diagnostically decisive. Molecular confirmation of the JAZF1-SUZ12 translocation provides additional diagnostic certainty in ambiguous cases [12,27].

In our case, the final pathology of the hysterectomy specimen showed no ESS within the uterine corpus, confirming true primary EESS without a uterine origin.

#### 3.2.3. Treatment

Surgical resection is the cornerstone of LG-EESS. For patients with an intact uterus and adnexa, total hysterectomy with BSO combined with excision of all macroscopic extrauterine diseases constitutes the standard surgical approach in alignment with the guidelines for uterine LG-ESS [9,10,23]. The rationale for BSO is compelling: Dai et al. [10] demonstrated that ovarian preservation was associated with a 4.9-fold (95% confidence interval [CI]: 2.3–10.1) increase in hazard of recurrence in primary LGESS, and a 3.1-fold (95% CI: 1.3–7.3) increase in hazard of repeated recurrence in recurrent disease, without a corresponding benefit to overall survival. In the present case, the patient had already undergone gastrointestinal resection (partial gastrectomy and colectomy) at the referring institution, and LESS total hysterectomy with BSO was performed to eliminate all residual hormonally responsive tissues. The use of an LESS approach enabled definitive gynecological surgery with minimal abdominal access in a patient who had recently undergone abdominal surgery, demonstrating the feasibility and safety of minimally invasive techniques in this setting [22,23].

Given the endocrine-dependent nature of the tumor, adjuvant hormonal therapy, most commonly with aromatase inhibitors (AI) or progestins, is recommended for patients with ER/PR-positive tumors, residual disease, or tumor [6,9,15,17,23]. Wu et al. [9] reported that adjuvant hormonal therapy significantly reduced recurrence in patients with LG-EESS who underwent optimal cytoreduction. Rajaram et al. [23] also documented favorable outcomes with GnRH analog therapy in fertility-preserving cases of uterine LG-ESS; however, uterine preservation in EESS remains under investigation.

Adjuvant hormonal therapy in LG-ESS has evolved considerably over the past decade. While progestins (medroxyprogesterone acetate and megestrol acetate) were historically the most widely used agents given the near-universal PR positivity of LG-ESS [8,9,10], current evidence and expert consensus favor aromatase inhibitors (AIs)—such as letrozole or anastrozole—as the preferred first-line adjuvant hormonal strategy, particularly following bilateral salpingo-oophorectomy [23,26]. AIs suppress residual peripheral estrogen synthesis, thereby depriving ER-positive tumor cells of their primary mitogenic stimulus, and have demonstrated superior efficacy over progestins in reducing recurrence risk in both uterine and extrauterine LG-ESS. Estrogen receptor blockade with fulvestrant represents an additional option, particularly in cases of AI intolerance or resistance. In the present case, adjuvant hormonal therapy discussions were initiated following surgery, and the choice of agent was individualized based on the patient’s ER/PR receptor status, menopausal state following BSO, comorbidities—including her underlying autoimmune thyroiditis and intracranial aneurysm—and patient preference. We acknowledge that explicit documentation of the adjuvant therapy decision-making process was insufficiently detailed in the original manuscript, and we have revised the Follow-up section accordingly to reflect the treatment considerations and the chosen hormonal regimen.

Chemotherapy has limited efficacy in LG-ESS and is reserved for refractory or rapidly progressive disease [9,26]. Radiotherapy may be considered in select cases with local recurrence or positive surgical margins.

#### 3.2.4. Prognosis

The prognosis of LG-EESS is generally favorable, although marked by a high propensity for late recurrence, sometimes decades after the initial diagnosis [3,8,15]. Ayuso et al. [3] reported a case of colonic EESS diagnosed 37 years after a hysterectomy for endometriosis, illustrating the extreme chronological latency of the disease. Wu et al. [9] reported a 10-year disease-free survival rate of 70% and a 10-year overall survival rate of 100% in LG-EESS, with recurrence occurring exclusively in patients who did not undergo optimal surgery or received hormonal therapy. Fukunaga et al. [5] reported no evidence of disease in any of the three patients with EESS at 16–39 months post-surgical resection. Singhal et al. [21] reported a disease-free survival of 65 months following complete cytoreductive surgery for vaginorectal EESS. Conversely, Park et al. [26] reported a fatal outcome in a patient with EESS arising from extensive pelvic endometriosis treated with suboptimal cytoreduction and systemic chemotherapy, underscoring the critical importance of surgical completeness. A systematic review by Deb and Heller [8] identified tumor site and treatment modality as the strongest predictors of both overall and progression-free survival, whereas age at diagnosis did not significantly influence the outcomes. Based on available evidence, long-term surveillance with clinical examination and periodic imaging is mandatory for all LG-EESS patients, given the risk of recurrence many years or decades after treatment [22].

### 3.3. Future Perspectives

Despite the growing number of reported EESS cases enabled by international case sharing and systematic reviews, several critical knowledge gaps remain. First, the molecular landscape of LG-EESS beyond the JAZF1-SUZ12 fusion is underexplored. Recent advances in transcriptomic and proteomic profiling of uterine LG-ESS have identified additional driver alterations (e.g., *JAZF1-PHF1*, *EPC1-PHF1*, and *MEAF6-PHF1* fusions) whose prevalence and prognostic significance in EESS remain unknown [8,27].

Second, the endometriosis-to-ESS transformation pathway remains poorly characterized at the mechanistic level. Elucidating specific molecular events, including the potential roles of chronic inflammation, estrogen receptor signaling, epigenetic dysregulation, and miRNA cargo, could identify targetable vulnerabilities and enable earlier detection in high-risk patients with endometriosis [18,22,26]. Third, standardized treatment algorithms for LG-EESS are lacking; current management is largely extrapolated from uterine ESS guidelines and small case series, and prospective multicenter registries are urgently needed to establish evidence-based protocols for surgical extent, adjuvant therapy selection, and surveillance interval [8,9,10]. Fourth, the role of fertility-preserving surgery in young patients with LG-EESS—while reported for uterine LG-ESS [6]—has not been systematically evaluated in the extrauterine setting, and the oncologic safety of deferring hysterectomy in this population remains undefined. Finally, the increasing adoption of minimally invasive surgery, including LESS and robotic-assisted approaches, to manage LG-EESS warrants prospective evaluation. Although technical feasibility has been demonstrated, comparative data on oncologic outcomes relative to open surgery are lacking [16,19,28]. As the case presented here illustrates, multidisciplinary coordination integrating gynecologic oncology, colorectal surgery, and pathology is essential for the optimal management of gastrointestinal EESS and should be formalized in future clinical guidelines.

The absence of a universally accepted staging system for primary EESS remains a critical gap; clinicians currently apply frameworks designed for either uterine sarcomas or general soft tissue sarcomas by analogy, neither of which fully captures the unique characteristics of extrauterine endometrial stromal tumors [8]. International multicenter collaboration to develop a consensus staging system for EESS, incorporating tumor site, size, adjacent organ invasion, nodal status, and hormone receptor expression, is strongly recommended to standardize prognostic stratification and facilitate future clinical trials.

### 3.4. Limitations

The relatively short follow-up duration available at the time of manuscript submission precludes definitive conclusions regarding long-term recurrence risk in this specific case; however, given the indolent yet recurrence-prone natural history of LG-ESS documented in the literature [4,8,26], continued surveillance remains mandatory regardless of short-term disease-free status.

The immunohistochemical panel applied to the primary gastrointestinal resection specimen at the referring institution was limited, and additional diagnostically informative markers—including SMA, h-caldesmon, cyclin D1, and BCOR—were not reported, which represents a limitation in the completeness of diagnostic characterization of this case.

## 4. Conclusions

This case report presents a rare case of primary LG-EESS involving the stomach and transverse colon in a 49-year-old female who underwent gastrointestinal resection at the referring institution, followed by LESS total hysterectomy and BSO at our center. The final pathological examination confirmed the absence of uterine ESS, establishing it as a true primary extrauterine tumor. Gastrointestinal LG-EESS is a rare variant of an uncommon malignancy, and the present case represents one of the very few reports of simultaneous gastric and colonic involvement. This case highlights the following three key clinical lessons: (1) LG-EESS should be included in the differential diagnosis of any LUQ or gastrointestinal mass in a female of reproductive or perimenopausal age, particularly in the setting of endometriosis or prominent parauterine vascularity on imaging; (2) immunohistochemical profiling with a broad panel—including CD10, ER, PR, beta-catenin, CD117, and desmin—is essential for accurate diagnosis and differentiation from GIST and smooth muscle tumors; and (3) complete surgical resection, including hysterectomy and BSO when applicable, combined with long-term surveillance, represents the optimal management strategy. Given the documented risk of late recurrence extending decades beyond initial treatment, indefinite follow-up is warranted. Future multicenter collaborative registries and molecular studies are needed to better characterize the biology, refine treatment algorithms, and improve the outcomes of patients with this rare gynecologic malignancy.

## Figures and Tables

**Figure 1:**
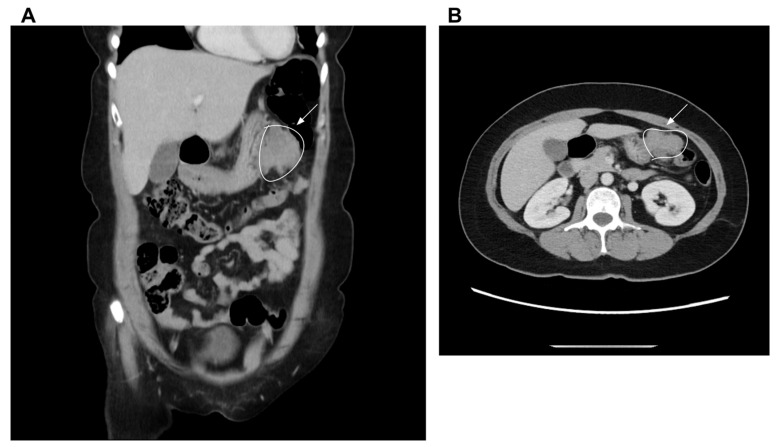
Abdominal computed tomography (CT) revealed a lobulated mass lesion between the LUQ abdomen (gastric greater curvature) and the splenic flexure of the colon, measuring at least 4.6 cm (arrow). (**A**) Coronal view, (**B**) Axial view.

**Figure 2:**
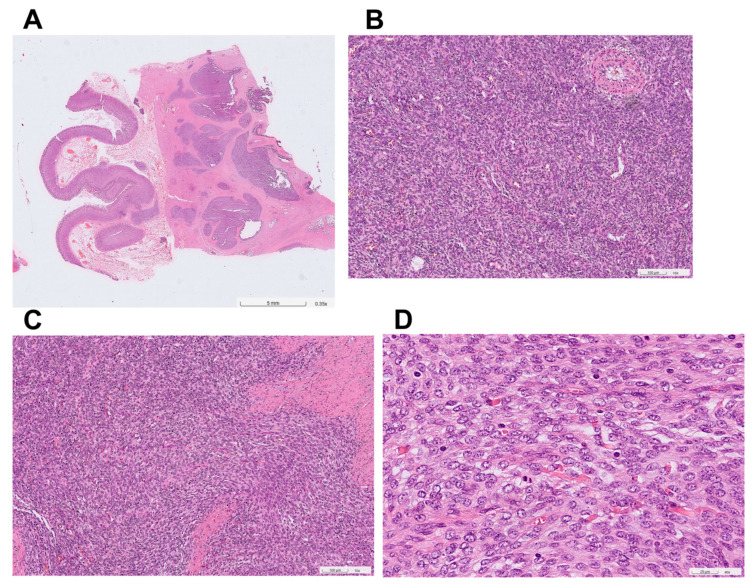
Histopathological features of extrauterine low-grade endometrial stromal sarcoma located between the greater curvature of the stomach and the splenic flexure of the colon. (**A**) Low-power overview (scale bar = 5 mm) demonstrating the excised extrauterine tumor mass with tongue- and worm-like infiltrative growth pattern of neoplastic stromal cells, with preservation of the surrounding architectural framework at the intra-abdominal implantation site between the greater curvature of the stomach and the splenic flexure of the colon. (**B**) Intermediate magnification (scale bar = 100 µm) showing sheets of uniform, densely packed small oval-to-spindle cells with scant cytoplasm arranged around a rich network of small arterioles closely resembling normal proliferative-phase endometrial stroma, consistent with extrauterine stromal implantation. (**C**) Intermediate magnification (scale bar = 100 µm) illustrating infiltrative stromal nodules with intervening fibrous bands, highlighting the permeative growth pattern characteristic of LG-ESS at this ectopic intra-abdominal location. (**D**) High-power view (scale bar = 25 µm) revealing uniform round-to-ovoid nuclei with finely dispersed chromatin, mild nuclear atypia, and inconspicuous mitotic activity, confirming the bland cytomorphology characteristic of LG-ESS and excluding high-grade transformation at this extrauterine site.

**Figure 3:**
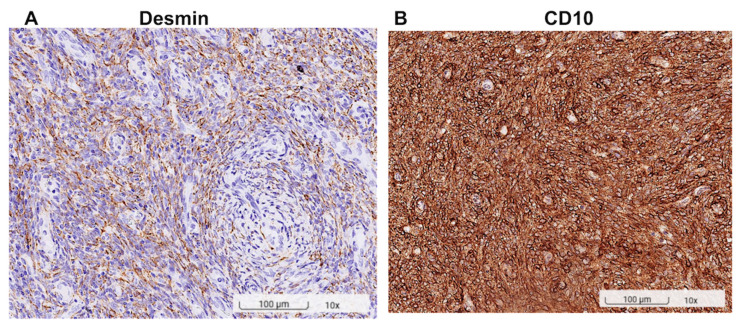
Immunohistochemical profile of extrauterine low-grade endometrial stromal sarcoma (LG-ESS). (**A**) Desmin immunostaining (scale bar = 100 µm) demonstrates focal and patchy cytoplasmic positivity predominantly within perivascular smooth muscle elements and scattered stromal cells, while most neoplastic stromal cells remain negative, consistent with the expected desmin-negative phenotype of LG-ESS and supporting distinction from smooth muscle neoplasms. (**B**) CD10 immunostaining (scale bar = 100 µm) showing diffuse and strong membranous and cytoplasmic positivity throughout the neoplastic stromal cell population, confirming endometrial stromal differentiation. The characteristic CD10-positive, desmin-negative immunophenotype, together with the morphological features, supports the diagnosis of LG-ESS.

**Figure 4:**
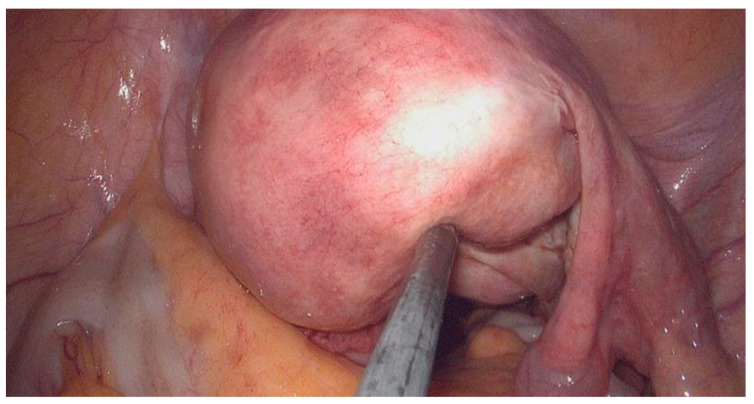
Laparoscopic view showed an enlarged uterus with a myoma and normal-appearing bilateral adnexa.

**Table 1:** Summary of reported cases of extrauterine and uterine low-grade endometrial stromal sarcoma (LG-ESS) from the reviewed literature.

No.	First Author (Year)	Journal	Age (yr)	Site	Endometriosis	Surgery	Adjuvant Therapy	Outcome
1	Tang et al. (2020) [2]	Int J Gynecol Pathol	74	Vagina	NR	Partial vaginectomy	None	NED 46 mo
2	Clair et al. (2017) [4]	Gynecol Oncol Rep	42	Omentum	Yes	Surgical resection	Hormone therapy	NED
3	Mercier et al. (2021) [7]	Gynecol Oncol Rep	53	Adnexa/distal ureter	NR	Surgical resection	NR	NR
4	Vierhout et al. (1992) [15]	Eur J Obstet Gynecol Reprod Biol	35	Extrauterine (NOS)	Yes	Surgery + chemo + hormone	Chemo + hormone	Disease course described
5	Kundu et al. (2020) [12]	Gynecol Oncol Rep	37	Colon	NR	Surgical resection	NR	NED
6	Rajaram et al. (2024) [23]	Gynecol Oncol Rep	22, 19	Uterine (fertility-sparing)	No	Uterine-sparing resection	GnRH analogue	NED
7	Tenjimbayashi et al. (2025) [16]	Cureus	52	Ovary	Yes	TH + BSO + omentectomy + rectal resection	MPA 600 mg/day	NED 3 yr
8	Ayuso et al. (2013) [3]	Case Rep Obstet Gynecol	80	Colon	Yes (hx)	Laparoscopic LAR	Megestrol acetate	NED > 4 yr
9	Zaza et al. (2015) [24]	Hematol Oncol Stem Cell Ther	50	Vulva	No (hx hysterectomy)	Local excision	Megestrol acetate	NED 28 mo
10	Garg et al. (2009) [17]	Rare Tumors	45	Rectovaginal septum	NR	Surgery	Hormone therapy	Response to hormone Tx
11	Lipsich et al. (2020) [18]	Clin Imaging	45	Ovary/peritoneum/pelvis	Yes	Right adnexectomy + debulking	NR	NR
12	Levine et al. (2001) [19]	Int J Gynecol Pathol	37	Extrauterine (ovarian/peritoneum)	Yes	Surgical resection	NR	NR
13	Talayeh et al. (2022) [6]	J Obstet Gynecol Cancer Res	20	Uterine	No	TAH + BSO	NR	NR
14	Djatikusumo et al. (NR) [25]	Inajog	44	Uterus + ovary + omentum + jejuno-ileum	No	Resection + staging	NR	No abnormality 5 mo post-op
15	Manjula et al. (2022) [11]	Arch Med Health Sci	48	Omentum	No	Surgical resection	NR	Asymptomatic 2 yr
16	Son et al. (2015) [13]	Ann Coloproctol	52	Sigmoid colon	NR	Laparoscopic LAR; later TH + BSO	NR	NR
17	Fukunaga et al. (1998) [5]	Pathol Int	40, 43, 50	Ovary/pelvic & abdominal cavity	Yes (2/3)	Surgical resection	NR	NED 16–39 mo
18	Cao et al. (2020) [14]	Eur J Gynaecol Oncol	46	Sigmoid colon (later uterine)	No	Laparoscopic sigmoid resection; later TH + BSO	None initially	Recurrence 2 yr later
19	Park et al. (2008) [26]	Korean J Gynecol Oncol	37	Pelvic/retroperitoneal (EESS)	Yes	Cytoreductive surgery	IP chemo + Megace	Expired 16 mo
20	Chen et al. (2005) [20]	Taiwan J Obstet Gynecol	28	Ovary (arising from endometriosis)	Yes	Left SO	High-dose progesterone	NR
21	Gorostiaga et al. (2021) [27]	Rev Esp Patol	75	Peritoneum (multiple implants)	No	Surgical resection	NR	Aggressive behavior noted
22	Singhal et al. (2023) [21]	Korean J Clin Oncol	38	Vagina/pouch of Douglas/rectum	Yes	Retrograde TH + posterior vaginal wall excision + rectosigmoid resection	NR	NED 65 mo
23	Dai et al. (2024) [10]	Int J Surg	Various	Uterine vs. extrauterine (cohort)	NR	Surgery (cohort study)	Hormone therapy	Similar OS; ovarian preservation increases recurrence
24	Haghighatkhah et al. (2024) [1]	Int J Cancer Manag	35	Peritoneum (stage IV endometriosis)	Yes	NR (biopsy confirmed)	NR	NR
25	Wu et al. (2022) [9]	World J Surg Oncol	Various	Omentum (most), ovary, vagina	NR	Cytoreductive surgery (10 cases)	Chemo + hormone (ER/PR+)	OS 100% at 5 & 10 yr; DFS 70%
26	Deb & Heller (2022) [8]	Ann Diagn Pathol	Various	Multiple sites	NR	Systematic review	Various	Tumor site & treatment affect OS & PFS
27	Schiestl et al. (2025) [22]	Gynecol Oncol Rep	31	DIE-associated extrauterine	Yes	Surgery	NR	NED; long-term surveillance needed
28	Li et al. (2026) (current report)	Diagnostics	49	Between gastric and colon	No	Resection of primary tumor, and LESS TH and BSO	Hormone	NED

Abbreviations: NED, no evidence of disease; NR, not reported; TH, total hysterectomy; BSO, bilateral salpingo-oophorectomy; SO, salpingo-oophorectomy; LAR, low anterior resection; TAH, total abdominal hysterectomy; MPA, medroxyprogesterone acetate; DIE, deep infiltrating endometriosis; IP chemo, intraperitoneal chemotherapy; OS, overall survival; PFS, progression-free survival; DFS, disease-free survival; EESS, extrauterine endometrial stromal sarcoma, LESS: laparoendoscopic single-site. Note: The substantial number of ‘not reported’ (NR) entries across multiple fields reflects the inherent heterogeneity and inconsistency of reporting in the published LG-EESS literature, which largely comprises single case reports without standardized data collection, underscoring the need for prospective multicenter registries and consensus reporting guidelines.

## Data Availability

The original contriubtion presented in this study are included in the article.

## Abbreviations

The following abbreviations are used in this manuscript:

LG-ESS	Low-grade endometrial stromal sarcoma
EESS	Extrauterine ESS
LESS	laparoendoscopic single-site
ESS	Endometrial stromal sarcoma
LUQ	left upper quadrant
CT	computed tomography
BSO	bilateral salpingo-oophorectomy
LG-EESS	low-grade extrauterine endometrial stromal sarcoma
ER	estrogen receptor
PR	progesterone receptor
GIST	gastrointestinal stromal tumor
